# Drought risk assessment under climate change is sensitive to methodological choices for the estimation of evaporative demand

**DOI:** 10.1371/journal.pone.0174045

**Published:** 2017-03-16

**Authors:** Candida F. Dewes, Imtiaz Rangwala, Joseph J. Barsugli, Michael T. Hobbins, Sanjiv Kumar

**Affiliations:** 1 Cooperative Institute for Research in Environmental Sciences, University of Colorado, Boulder, Colorado, United States of America; 2 Physical Sciences Division, NOAA/ESRL, Boulder, Colorado, United States of America; 3 Department of Interior North Central Climate Science Center, Fort Collins, Colorado, United States of America; University of Vigo, SPAIN

## Abstract

Several studies have projected increases in drought severity, extent and duration in many parts of the world under climate change. We examine sources of uncertainty arising from the methodological choices for the assessment of future drought risk in the continental US (CONUS). One such uncertainty is in the climate models’ expression of evaporative demand (E_0_), which is not a direct climate model output but has been traditionally estimated using several different formulations. Here we analyze daily output from two CMIP5 GCMs to evaluate how differences in E_0_ formulation, treatment of meteorological driving data, choice of GCM, and standardization of time series influence the estimation of E_0_. These methodological choices yield different assessments of spatio-temporal variability in E_0_ and different trends in 21^st^ century drought risk. First, we estimate E_0_ using three widely used E_0_ formulations: Penman-Monteith; Hargreaves-Samani; and Priestley-Taylor. Our analysis, which primarily focuses on the May-September warm-season period, shows that E_0_ climatology and its spatial pattern differ substantially between these three formulations. Overall, we find higher magnitudes of E_0_ and its interannual variability using Penman-Monteith, in particular for regions like the Great Plains and southwestern US where E_0_ is strongly influenced by variations in wind and relative humidity. When examining projected changes in E_0_ during the 21^st^ century, there are also large differences among the three formulations, particularly the Penman-Monteith relative to the other two formulations. The 21^st^ century E_0_ trends, particularly in percent change and standardized anomalies of E_0_, are found to be sensitive to the long-term mean value and the amplitude of interannual variability, i.e. if the magnitude of E_0_ and its interannual variability are relatively low for a particular E_0_ formulation, then the normalized or standardized 21^st^ century trend based on that formulation is amplified relative to other formulations. This is the case for the use of Hargreaves-Samani and Priestley-Taylor, where future E_0_ trends are comparatively much larger than for Penman-Monteith. When comparing Penman-Monteith E_0_ responses between different choices of input variables related to wind speed, surface roughness, and net radiation, we found differences in E_0_ trends, although these choices had a much smaller influence on E_0_ trends than did the E_0_ formulation choices. These methodological choices and specific climate model selection, also have a large influence on the estimation of trends in standardized drought indices used for drought assessment operationally. We find that standardization tends to amplify divergences between the E_0_ trends calculated using different E_0_ formulations, because standardization is sensitive to both the climatology and amplitude of interannual variability of E_0_. For different methodological choices and GCM output considered in estimating E_0_, we examine potential sources of uncertainty in 21^st^ century trends in the Standardized Precipitation Evapotranspiration Index (SPEI) and Evaporative Demand Drought Index (EDDI) over selected regions of the CONUS to demonstrate the practical implications of these methodological choices for the quantification of drought risk under climate change.

## Introduction

Drought is a major climatic phenomenon affecting socio-ecological systems worldwide through scarcity of available water [1,2]. This scarcity generally arises from a sustained and extended period of precipitation deficiency, such as in the 2012–2016 California drought, but it often is initially set off and further intensified by increased water demand by the atmosphere and society [3–5]. In this paper we focus on atmospheric evaporative demand (i.e., “the thirst of the atmosphere”), which represents the amount of water that would evaporate from the earth’s surface and be transpired by plants if water availability were not a limiting factor (e.g., [6]).

Future drought risks are expected to change due to changes in the hydroclimatological drivers of drought processes [7]; these include changes in large-scale moisture transport [8], precipitation patterns [9], snow processes [10,11], and increases in evaporative demand (E_0_) in a warmer atmosphere and its influence on soil moisture [12,13]. Previous studies have warned of increases in drought severity, extent, and duration, affecting the water balance in many parts of world under climate change [14,15], including regions in the continental US (CONUS; [16,17]). These projection studies typically focus on either the precipitation deficit itself or some proxy of soil water availability. Societal impact studies also factor in increasing water demand by agriculture and other human activities [18–20]. On the other hand increased aridity from enhanced E_0_ will likely be partially offset by increased water-use efficiency in plants due to elevated atmospheric CO_2_ concentrations [21–23].

Recently, there has also been a concerted effort to frame drought risk in the context of ecosystem impacts, where an episodic deficit in “ecologically available water” can potentially drive ecosystems beyond thresholds of vulnerability (e.g., widespread tree mortality, species habitat shifts), often with cascading impacts in coupled natural-human systems. In an effort to incorporate important ecological impacts that do not fit into existing drought definitions (i.e., meteorological, hydrological, or agricultural droughts [24,25]) and to pave the way for new policies and adaptive frameworks, the Science for Nature and People Partnership [26] have emphasized the concept of “Ecological Drought” [27,28], which they define as “a prolonged and widespread deficit in naturally available water supplies—including changes in natural and managed hydrology—that create multiple stresses across ecosystems”. In this context, natural resource managers and decision makers must anticipate future drought risk and assess the uncertainty associated with projected changes as they develop regional drought plans [29]. Part of the uncertainty in drought risk assessments arises from methodologies employed in the study, which include the choice of drought metrics, the methodology to estimate the various physical terms in those metrics (evaporative demand here), and uncertainties in global climate model (GCM) projections at regional scales [30–34].

Of particular concern related to changing drought risk under climate change is the increase in E_0_ due to a warmer atmosphere, which could potentially enhance drying and heating of the land surface, causing more frequent, long-lasting, and more severe soil moisture depletions [15,35]. E_0_ is both a driver of drought (i.e., it controls loss of water from the land surface) and an indicator of drought (through the complementary relationship between actual ET and E_0_, e.g.[36]). If properly estimated, E_0_ is sensitive to physical variables such as net radiation, temperature, humidity, and wind speed, and it both influences and is influenced by soil moisture-atmosphere feedbacks. Furthermore, E_0_ is extensively considered for agricultural and ecological impacts assessments, and many operational drought indicators (e.g., PDSI, SPEI, ESI, EDDI) use this term.

Recent studies [21,37] suggest that E_0_-based drought indicators can overestimate future drought predictions by not incorporating plants’ physiological response to elevated CO_2_ concentrations, i.e., and increased water-use efficiency that leads to a similar carbon uptake and photosynthesis for lower transpiration losses from the plant. As such the impacts of changes in E_0_ in climate projections should be interpreted with care. However, for the purposes of this paper, the use of a standard reference serves to isolate the methodological issues considered. Further, GCM generally have coarse spatial resolutions and land surface responses show significant uncertainties [38–40]. The assessment of E_0_ is therefore very instructive, particularly when we examine hydro-climatic processes at finer temporal and spatial resolutions relevant to regional impacts assessment, as well as taking the benefit of the more widely available downscaled GCM output of atmospheric variables.

Potential evaporation and reference evapotranspiration are widely used measures of E_0_. Historically, several different formulations ranging from simple temperature-based formulae to the more complex physically based methods (i.e., those that incorporate all the physical drivers—radiative and advective—that affect the energy balance that would hold over a wet surface in given climatic conditions) such as the Penman-Monteith [41], have been used to estimate this term (e.g., [42,43]). Even within a single method, different choices of parameter estimation exist, e.g. Scheff and Frierson [44] found that Penman-Monteith-based E_0_ estimation shows 10–30% variation across the temporal scales of the input data (3-hourly versus monthly). Similarly, in the Penman-Monteith formula, different studies have used either short- or tall-crop coefficients (discussed later) which can considerably affect E_0_ estimation [44–46]. While E_0_ estimation remains an active research area (e.g., [21,37]) there is no clear set of guidelines or recommendations for user communities interested in assessing and quantifying climate change-induced drought impacts at regional scales. For example, the U.S. Department of Interior’s North-Central Climate Science Center is interested in better understanding the future drought trends in the Northern Great Plains region [47], in order to properly inform stakeholders and resource managers in their adaptation plans. E_0_ is also an important variable for ecological applications, yet a number of habitat, species-distribution, and vegetation models still use simplified radiation-based methods to estimate E_0_ while investigating impacts of climate change on terrestrial ecosystems (e.g., [48–50]). Another example is the commonly used Aridity Index (AI; [51]) for the assessment of future drought risk. While some studies employ the AI computed with physically based E_0_ (e.g., [52,53]), many others rely on AI computed using temperature-based methods such as Hargreaves-Samani [54,55] to assess impacts of climate change on ecosystems (e.g., [22,31,56]).

A primary goal of this study is to evaluate how some widely used E_0_ formulations differ in their estimation of E_0_, including in its spatial and temporal variabilities and trends under climate change. Furthermore, we also investigate how different choices of parameters and temporal scale of input data for a specific formulation affect E_0_ estimation and future trends. Previous studies have done cross-comparisons of different E_0_ formulations (e.g., [1,57–60]), but only a few studies (e.g., [61,62]) have examined the importance of methodological choices for drought risk assessment at regional scales under climate change scenarios, in particular for the CONUS. For example, Sheffield et al. [1] found that 20^th^ century global drying trends differ between temperature- and physically based E_0_ estimation methods. A more exhaustive comparison including several E_0_ formulations, choices of parameter estimations, and drought metrics is needed for regional application in drought analyses. Here we compare such uncertainties coming from the methodological choices to estimate E_0_ (use of different formulations and input data), and selection of GCM output for the CONUS (see Table 1). Another focus of this study is how “standardized” drought indices are affected by different methodological choices. Standardized drought indices are widely used operationally because they allow comparisons of drought conditions across space and time (e.g., [63]). E_0_ is central to the calculation of a variety of commonly used drought indices, which include the formerly mentioned Aridity Index, the Palmer Drought Severity Index (PDSI; [64]), the Standardized Precipitation Evapotranspiration Index (SPEI; [65]), and the recently developed Evaporative Demand Drought Index (EDDI; [36,66]). Differences in the estimation of E_0_ from different methodologies will affect how these metrics represent the magnitude, duration, and the spatial extent of drought in the historical climate and in projections of future drought (e.g., [1]). Therefore, we also evaluate how future trends in standardized E_0_ anomalies and trends in standardized drought indices like SPEI and EDDI are sensitive to methodological and GCM choices made when estimating E_0_. This is crucial because ecologists and natural resource managers have a wide variety of methods and GCM datasets to choose from, but generally rely on limited guidance in the literature as to how sensitive their findings could be to these choices. This paper addresses this problem by comparing different methodologies for E_0_ estimation, two drought indicators (EDDI and SPEI), and investigating their impacts on present and future droughts conditions at regional scales within the CONUS.

**Table 1:** Analysis summary.

E_0_ Formulations (Drivers)	Input Data Choices (Penman-Monteith only)	GCM Selection
Hargreaves-Samani (temperature)	monthly versus daily wind speeds	
		low versus high climate sensitivity
Priestley-Taylor (temperature and radiation)	tall versus short crop	
		cold/wet versus hot/dry (model bias relative to the observed climate)
Penman-Monteith (temperature, radiation, humidity and wind speed)	GCM versus parameterized radiation terms	

We evaluate 21^st^ century trends in evaporative demand (E_0_) and standardized drought indices (SPEI and EDDI) that use E_0_ as function of the methodological choices summarized above.

## Methods: Estimating E_0_ and future drought risk

### Data

**Fig 1:**
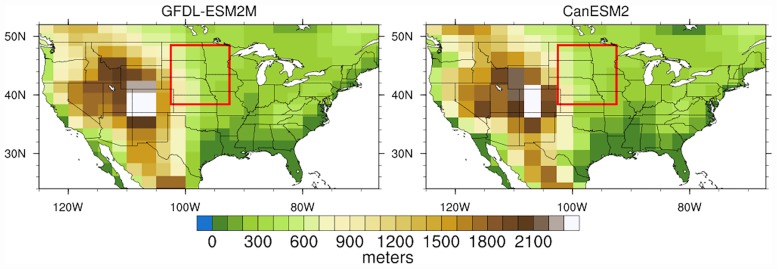
The topography of the CONUS in GFDL-ESM2M (left) and in CanESM2 (right). The red boxes delineate the Northern Great Plains region adopted in this study, over which E_0_ is spatially averaged at each daily time step.

We use daily outputs from the Coupled Model Intercomparison Project 5 (CMIP5) Historical (1950–2005) and Representative Concentration Pathway 8.5 (RCP8.5, 2006–2100) climate simulations from two GCMs: GFDL-ESM2M and CanESM2. Model specifications and their climate sensitivities are shown in Table 2 (sensitivity values given by Forster et al. [67]), and the topography of the CONUS in each model is depicted in Fig 1; more information about these models can be found in Flato et al. [68]. We use only the first ensemble member from each GCM’s simulations (only one member is available for GFDL-ESM2M) and we work with the data in their native grid resolutions. We select these models because of their availability of the required daily variables and their diverging biases and climate sensitivities. For example, in the Northern Great Plains region, GFDL-ESM2M has a cold (-1°C air temperature) and wet (+40% precipitation) bias while CanESM2 has a hot (+4°C) and dry (-10%) bias, with biases defined as the difference between the models’ historical climate and observations (where observations are drawn from University of Delaware v3.01 data, for temperature, and WMO’s Global Precipitation Climatology Centre v5 data, for precipitation). Furthermore, CanESM2 projects a 2°C greater warming by 2050 than GFDL-ESM2M for the Northern Great Plains (region shown by red boxes in Fig 1), although both models project similar increases in precipitation.

**Table 2:** Global climate model specifications and sensitivities.

Model	Modeling center	Resolution (degrees lon x lat)	Climate sensitivities (°C)	
			*ECS*	*TCR*
**CanESM2**	Canadian Centre for Climate Modeling and Analysis, Canada	2.8 x 2.8	3.69	2.40
*2*^*nd*^ *Generation Canadian Earth System Model*				
**GFDL-ESM2M**	NOAA/Geophysical Fluid Dynamics Laboratory, USA	2.5 x 2.0	2.44	1.30
*Geophysical Fluid Dynamics Laboratory Earth System Model with Modular Ocean Model component*				

*ECS* = Equilibrium Climate Sensitivity; *TCR* = Transient Climate Response.

To compute E_0_, we used daily maximum and minimum air temperature (*tasmax* and *tasmin*), relative humidity (*rhs*), wind speed (*sfcWind*), and upward and downward shortwave and longwave radiation (*rsus*, *rsds*, *rlus*, and *rlds*) at the surface. To compute the drought risk indices, we used our daily estimates of E_0_ and also the models’ daily precipitation rate (*pr*). The purpose of this paper is to illustrate how different methodologies can lead to very different projections of drought risk under climate change scenarios, therefore we restricted our analyses to two climate models. We do not extensively evaluate different plausible scenarios for future drought risk using several CMIP5 climate models, such as those done by Cook et al. [17] and Kumar et al. [46], for example.

Pan evaporation is an alternative measure of atmospheric evaporative demand for which long term and spatially distributed observations are available, and is closely related to E_0_ [69]. Pan evaporation observations are well-modeled by the PenPan equation, a version of the Penman method, indicating that physically-based Penman-type methods are able to capture the mean and variability of E_0_, including sensitivity to individual drivers [70–72]. We use station-based pan evaporation observations from the NOAA Cooperative Observer (COOP) Network to evaluate the ability of each E_0_ method to reproduce the spatial patterns of observed warm-season (defined here as May-September) E_0_ and its interannual variability. The data consists of an extension of that described in Hobbins [73,74]. That dataset was comprised of 228 stations reporting warm season (May-October) pan evaporation with at least 20 years of data between 1950 and 2001. Because here we define the warm season as May-September, we were also able to extend the dataset by adding 21 stations north of 41N latitude (see S1 Table for names and IDs of these stations). As noted in [73], the pan evaporation data require extensive quality control and homogenization due to documented and undocumented station moves and other factors. For the additional stations we followed the procedure in [73], including an adjustment in the mean when documented station moves occurred, and the removal of obvious outliers. We did not test for undocumented inhomogeneities for these stations. Instead, as in [73], further visual inspection of all station timeseries was used to add additional breakpoints in the data for homogenization (only two were added), and to eliminate two stations from consideration.

### Quantifying evaporative demand

Potential or reference evapotranspiration are widely used measures of E_0_. Potential evapotranspiration is the idealized flux of water from a surface to the atmosphere under unlimited moisture conditions (e.g., [6]), whereas reference evapotranspiration is the idealized flux of water from a reference crop under strictly specified surface and moisture conditions that lead to no moisture stress in plants (i.e., “well-watered”; [69]). We consider the following three formulations for estimating E_0_ because of their wide application both operationally and in research (e.g., [21,37,75]).

#### 1. Penman-Monteith formulation of reference evapotranspiration

This physically based estimate of E_0_ ([41,69,76,77]) is determined using the following equation:

$$E_{0}=\frac{0.408\;\Delta }{\Delta +\gamma \left(1+c_{d}u\right)}\left(R_{n}-G\right)+\frac{\gamma \frac{c_{n}u}{T+273}}{\Delta +\gamma \left(1+c_{d}u\right)}\;\left(e_{s}-e_{a}\right)$$ (1)

Here, the unit for E_0_ is mm day^-1^. The constant 0.408 (m^2^ mm MJ^-1^) represents the inverse of the latent heat of vaporization, *R*_*n*_ (MJ m^-2^ day^-1^) is the net radiation at the crop surface, *G* (MJ m^-2^ day^-1^) is the downward soil heat flux, *T* (°C) is the air temperature at a 2-m height, *u* (m s^-1^) is the wind speed at a 2-m height, *e*_*s*_ (kPa) is the saturation vapor pressure, *e*_*a*_ (kPa) is the actual vapor pressure (thus *e*_*s*_*−e*_*a*_ is the vapor pressure deficit), *Δ* (kPa °C^-1^) is the slope of temperature-saturation vapor pressure curve at *T*, *γ* is the psychrometric constant (kPa °C^-1^), *c*_*n*_ (K mm s^3^ Mg^-1^ day^-1^) and *c*_*d*_ (s m^-1^) are the numerator and denominator constants specific to reference crop type at the given time step (in this case, daily) [77]. *c*_*n*_ includes the effects of aerodynamic conductance that increases with vegetation height [78], while *c*_*d*_ represents a ratio of aerodynamic to leaf surface conductance (i.e., the inverse of surface resistance) that is likely to change under elevated CO_2_ concentration in the future [21,37] (this effect is not considered in this study). The first term on the right-hand side of Eq 1 is driven by the energy available for evaporation, which is given by *R*_*n*_ − *G*. The second term is driven by the ability to transport the water vapor away from the evaporating surface so that vapor pressure gradient is maintained; it is also known as the advective or aerodynamic term [79]. We computed each term using daily *tasmin*, *tasmax*, *rhs*, *sfcWind*, *rsus*, *rsds*, *rlus*, and *rlds* from the CMIP5 models. Following Allen et al. [69] (hereafter, FAO56), we primarily adopted the “tall crop” surface parameters (*c*_*n*_ = 1600 and *c*_*d*_ = 0.38), but a comparison with “short crop” parameters (*c*_*n*_ = 900 and *c*_*d*_ = 0.34) is also discussed. Note that a constant *c*_*d*_ value is adopted in this study, as the FAO56 method does not account for the effect of reducing *c*_*d*_ under elevated CO_2_ concentrations.

Because the concept of reference evapotranspiration was originally intended to represent small-scale field conditions, we test its transferability to the large-scale climate model world. Specifically, we assess the impact of parameter choices by computing variations in E_0_ responses from Eq 1 based on various input parameters for the radiative and aerodynamic terms in the formulation, and for different temporal scales of wind speed estimates:

1. Radiative inputs: we assessed the impact on E_0_ of net radiation (*R*_*n*_) taken from GCM outputs, as opposed to parameterization in the FAO56 method, where net radiation is computed using the GCM’s downward shortwave radiation and a fixed surface albedo of 0.23 while the net longwave radiation is parameterized (*PM-FAO56*). We compared this with E_0_ estimates using GCM outputs for upward and downward longwave radiation while keeping the same fixed surface albedo (*PM-gcmLW*), and also including all the radiative terms from the GCM (i.e., where albedo is variable, *PM-gcmSWLW*). We use this latter version (*PM-gcmSWLW*) to compare against the Hargreaves-Samani and the Priestley-Taylor formulations throughout this paper.
2. Reference crop type: we evaluated differences in E_0_ responses using the tall and short crop parameters described earlier.
3. Wind speed: we compared the use of daily wind speed inputs (as given by the GCMs) to the use of monthly mean wind speed (i.e., where daily values are made equal to monthly mean value). We chose to test this effect for wind speed alone because, among all drivers of E_0_, wind speed has the highest daily variability, and we hypothesized that using monthly means would thus underestimate the variability of E_0_.

#### 2. Hargreaves-Samani reference evapotranspiration

The FAO56 method recommends that, in the absence of all other meteorological data required for the Penman-Monteith equation, the temperature-based estimate of E_0_ proposed by Hargreaves and Samani [55] should be used. It takes the following form:

$$E_{0}=a\;R_{A}\;{\left(T_{max}-T_{min}\right)}^{0.5}\;\left(T_{avg}+17.8\right).$$ (2)

This formulation includes a radiation term, *R*_*A*_, which is top-of-the-atmosphere downward solar (extraterrestrial) radiation, here given in mm day^-1^, as is E_0_. However, *R*_*A*_ is derived from time of year and latitude only, so the Hargreaves-Samani method is considered temperature-based [80]. Another very commonly used temperature-based method for E_0_ estimation is the Thornthwaite method [81]. However, that method was originally proposed for monthly data, while our analyses focuses on daily estimates. Here we employed the GCMs’ daily *tasmax*, *tasmin*, and derived *T*_*avg*_ (in °C), and the value of *a* = 0.0023 (an adjustment constant) as described in FAO56.

#### 3. Priestley-Taylor potential evapotranspiration

This radiation-based method is more commonly used to estimate evaporative demand over extensive wet areas. It is a simplified form of the Penman equation [76], where the aerodynamic term is approximated to be 25 to 30% of the energy driven term (commonly set to 26%, or 1.26) [80,82], as follows:

$$E_{0}=1.26\;\frac{\Delta \;\left(R_{n}-G\right)}{\left(\Delta +\gamma \right)\;\lambda }$$ (3)

We used the GCM’s *tasmax* and *tasmin* to estimate *Δ* and *rsus*, *rsds*, *rlus*, and *rlds* fluxes to estimate *R*_*n*_. As in the Penman-Monteith method, *G* is assumed negligible at daily time scales.

Our analysis primarily focuses on the warm season which we select to be covering the period from May through September (hereafter MJJAS). Most of the annual E_0_ occurs in the warm season and thus, from an E_0_ perspective, it is the main period of concern for drought processes.

### Drought indices

We selected two operational drought-monitoring indices to quantify drought risk: the Evaporative Demand Drought Index (EDDI; [36,66]) and the Standardized Precipitation Evapotranspiration Index (SPEI; [65]). EDDI, which is an emerging drought index, is based solely on E_0_, while SPEI evaluates the balance between moisture supply (precipitation) and demand (evaporative demand) in the atmosphere (a balance we hereafter refer to as P—E_0_). Both indices can be computed for various time scales, and are intended to detect fast-evolving droughts (weeks to months) and characterize persistent seasonal and long-term droughts (months to years). While EDDI was originally developed for use with physically based E_0_, SPEI was originally developed with a temperature-based E_0_. Recent studies [83,84] have suggested that computing SPEI with a physically based formulation of E_0_ might be more appropriate to avoid overestimating the effect of increasing temperatures on E_0_. We compute 12-week (i.e., roughly a 3-month season) EDDI and SPEI using the three E_0_ formulations described above. Both indices are standardized relative to a 40-year climatology period (1966–2005).

We computed EDDI using the non-parametric approach described in Hobbins et al. [36], repeated here for the reader’s convenience. First, we computed empirical probabilities of E_0_ using the Tukey plotting position formula [85]. For each day between 1950–2100, daily E_0_ estimates were aggregated over the preceding 12 weeks, thus maintaining a daily temporal resolution in our timeseries. Next we ranked each 12-week E_0_ total against the 12-week totals for the same date in the years of the reference period (1966–2005). To give an example, the 12-week E_0_ for August 31 is the summation of daily E_0_ from June 9 to August 31, and we ranked the 12-week E_0_ for August 31 in 2036 against all the August 31 12-week E_0_s from the reference period. The empirical probabilities were then mapped to an inverse normal approximation [86] of these ranks to obtain standardized EDDI values. EDDI is distributed in a ~N(0,1) fashion (i.e., a standard normal distribution), with a range that is a function of the number of years in the reference period: in our case (n = 40) EDDI has a range of ±2.14. Because the distribution is normal, it can also be represented by percentiles. A zero EDDI value indicates that E_0_ accumulated over the aggregation period in a given year is equal to the median value from the reference period (the 50th percentile); negative values (lower E_0_, lower percentiles) indicate wet anomalies and positive values (higher E_0_, higher percentiles) indicate dry anomalies.

SPEI is an index of precipitation minus potential evapotranspiration (P-Eo). We computed 12-week SPEI using a modified version of the original procedure described in Vicente-Serrano et al. [65]. While the original study used a log-logistic distribution to compute the probabilities of P—E_0_ values at various scales exceeding the climatological median, their procedure did not account for seasonality and thus hindered the comparison to EDDI. In addition, when using daily aggregates of P—E_0_, we found the estimation of the parameters of the log-logistic distribution to be unstable. Therefore we adopted a non-parametric calculation procedure analogous to EDDI, as probability-based approaches allow for more consistent comparisons between standardized indices [87]. Just as described earlier for EDDI, daily P—E_0_ estimates were aggregated to continuous 12-week totals and ranked against the same-day 12-week accumulations within the reference period. SPEI values were determined from the empirical ranking probabilities relative to the reference period, also covering the range of ±2.14. In this case, positive SPEI values indicate wet anomalies while negative SPEI values indicate dry anomalies. As with EDDI, the range of SPEI values can also be represented by percentiles.

## Results

### E_0_ responses compared between Penman-Monteith, Hargreaves-Samani and Priestley-Taylor formulations

**Fig 2:**
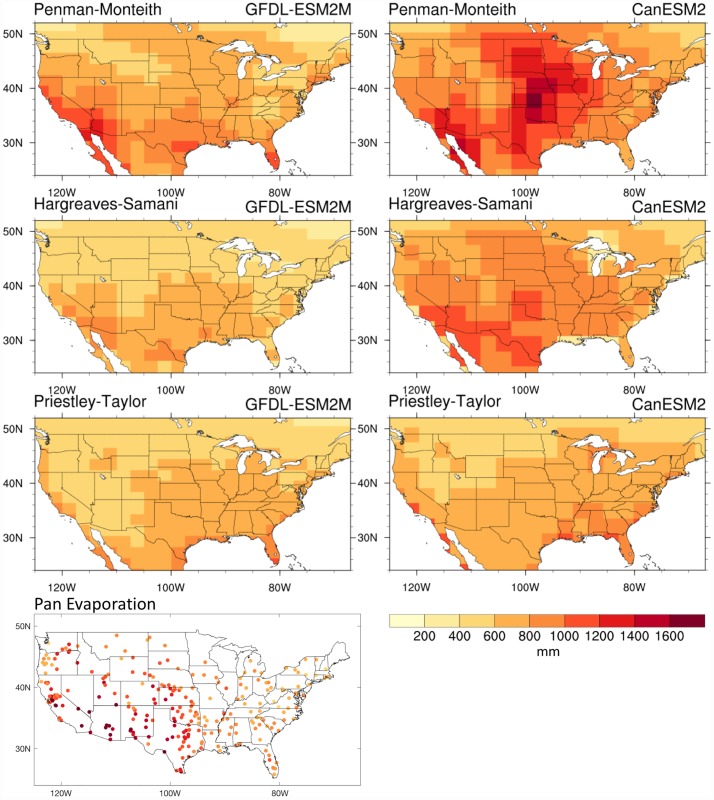
Historical mean E_0_ across the different E_0_ formulations. Climatological mean E_0_ (mm) across the CONUS for the MJJAS period in GFDL-ESM2M (first three plots in left column) and CanESM2 (right column) as estimated by the Penman-Monteith (first row), Hargreaves-Samani (second row), and Priestley-Taylor (third row) formulations for the 1976–2005 period. The bottom left plot shows observed mean MJJAS pan evaporation across the CONUS from 228 stations which had at least 20 years of data between 1950 and 2001.

Historical climatologies (1976–2005) of E_0_ values across the CONUS for MJJAS, calculated from the three E_0_ formulations with driving variables drawn from the GFDL-ESM2M and CanESM2 models, are shown in Fig 2, along with the observed MJJAS mean pan evaporation. In general, all three methods show a decrease in E_0_ values with increasing latitude. However, the pattern is more spatially heterogeneous in the values estimated by Penman-Monteith. Most notable are the large magnitudes in the southwestern US and Great Plains region, which are less pronounced in Hargreaves-Samani and mostly absent in Priestley-Taylor. For both GCMs, the Penman-Monteith based E_0_ maps most closely resemble the spatial pattern of pan evaporation. We also find higher magnitudes of E_0_ in Penman-Monteith, and in particular for the regions that are strongly influenced by wind-driven turbulence and relative humidity like the southwestern US and the Great Plains (e.g., [5,72,88]). Wind speed and specific humidity are not adequately accounted for in the Hargreaves-Samani and Priestley-Taylor formulations.

**Fig 3:**
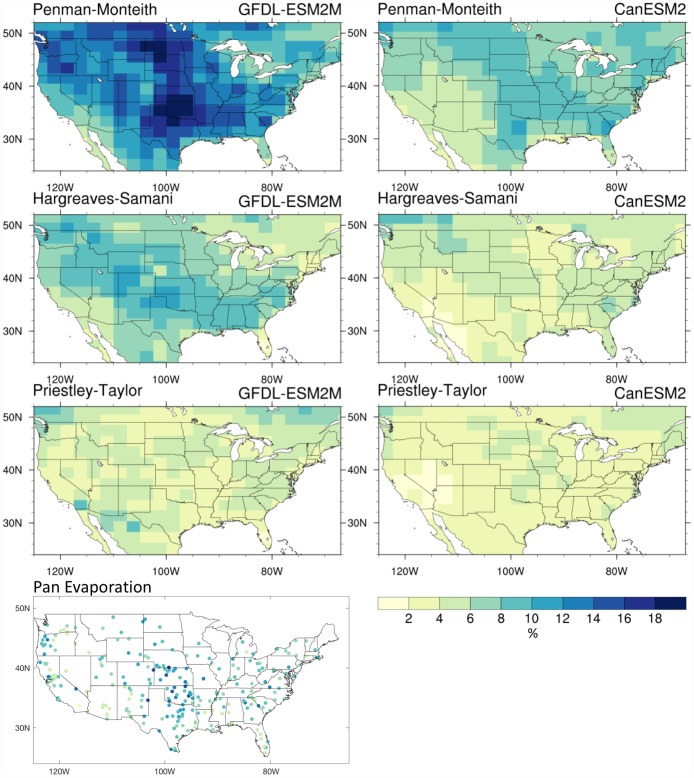
Historical coefficient of variation across the different E_0_ formulations. E_0_ coefficient of variation (CV, times 100 for %) across the CONUS for MJJAS in GFDL-ESM2M (first three plots in left column) and CanESM2 (right column) as estimated by the Penman-Monteith (first row), Hargreaves-Samani (second row), and Priestley-Taylor (third row) formulations for the 1976–2005 period. The bottom left plot shows the CV of observed MJJAS pan evaporation across the CONUS from 228 stations which had at least 20 years of data between 1950 and 2001.

In Fig 3 we compare the coefficient of variation (CV) in seasonal (MJJAS) E_0_ across the CONUS between the three formulations driven by the data from the two GCMs for the 1976–2005 period, including the CV of observed MJJAS pan evaporation. Here again, there are large differences between the three formulations, with Penman-Monteith maps depicting the largest CV magnitudes, particularly in the Great Plains region. The CV is indicative of year-to-year variability in seasonal E_0_, an accurate depiction of which is required to adequately capture drought risks. Station-based pan evaporation observations confirm the maximal CV over the Great Plains region as found in the Penman-Monteith formulation, which the other two formulations do not adequately capture, in particular the Priestley-Taylor formulation.

**Fig 4:**
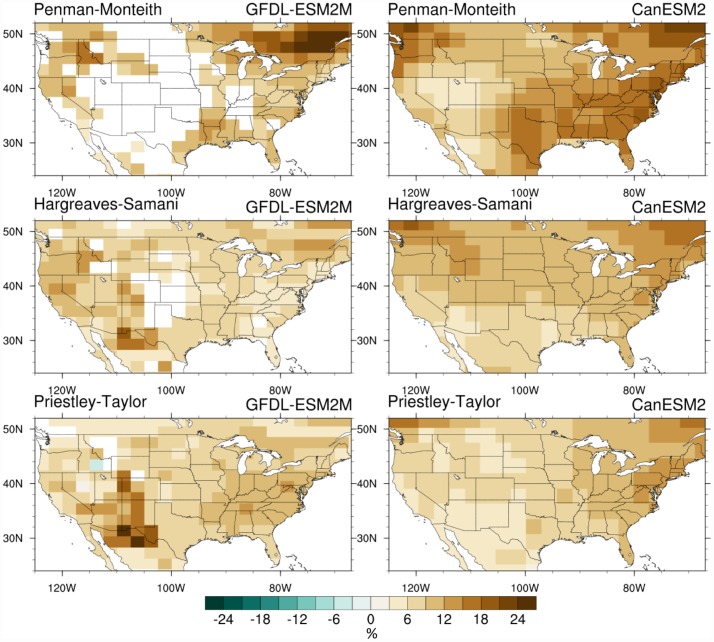
Projected changes (%) in E_0_ by 2050 across the different E_0_ formulations. Percent change in mean MJJAS E_0_ from Penman-Monteith (top row), Hargreaves-Samani (center), and Priestley-Taylor (bottom) formulations for GFDL-ESM2M (left) and CanESM2 (right) by 2050 (2036–2065) relative to the historical (1976–2005) period. Grid cells where the change is not statistically significant (i.e., p > 0.05) are masked out in white.

Next we investigate 21^st^ century projections in E_0_ between these three formulations. The primary intention here is less to evaluate what future E_0_ projections may look like and more to explore how different methodological choices affect these projected changes. First, we examine the future changes in E_0_ and their spatial variation across the CONUS. Fig 4 shows changes in mean MJJAS E_0_ by 2050 based on the three formulations as a percentage of the historical (1976–2005) mean. We use the t-test at 5% significance level to determine whether the projected change is statistically significant. These results show that, to the first order, the choice of GCM drives major differences in the projected changes. Overall, with the exception of one grid cell (in Priestley-Taylor based on GFDL-ESM2M), E_0_ increases by mid-21st century. CanESM2 shows significant increases in E_0_ across the whole domain and for all the three formulations, while GFDL-ESM2M generally shows more modest increases than CanESM2, with large regions in the central and western US where the change is not significant when using the Penman-Monteith formulation. Across the three formulations, these projected changes also differ between the GCMs. For example, Priestley-Taylor gives the largest CONUS-wide increase in GFDL-ESM2M, while Penman-Monteith has that for CanESM2.

**Fig 5:**
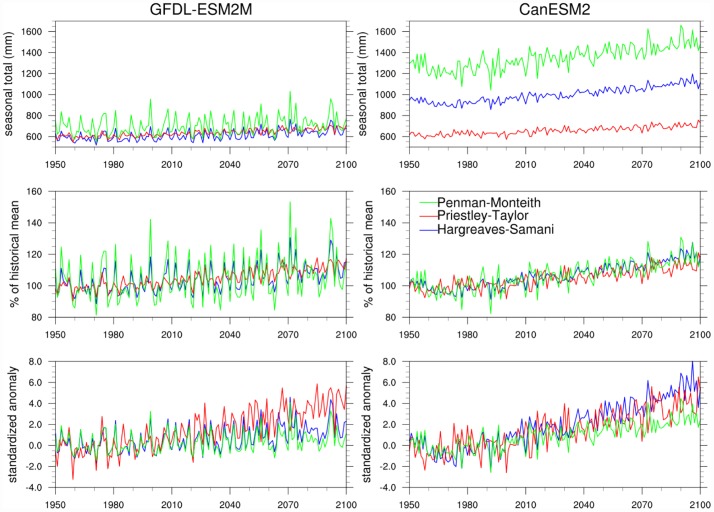
21^st^ century trends in E_0_ across the different E_0_ formulations. Trends in MJJAS E_0_ projected from Penman-Monteith (green), Hargreaves-Samani (blue), and Priestley-Taylor (red) formulations driven by GFDL-ESM2M and CanESM2 data for the Northern Great Plains. The top row shows seasonal totals in mm, center row shows E_0_ anomalies as % of the 1976–2005 mean, and bottom row shows standardized E_0_ anomalies.

Next, we examine and compare temporal trends in E_0_ between the three formulations for a selected region, i.e., the Northern Great Plains as delineated by the rectangular boxes in Fig 1. Fig 5 shows E_0_ trends for MJJAS in seasonal totals, percent of the 1976–2005 mean, and standardized anomalies based on the three formulations and the two GCMs. Here again, large differences are evident between the three formulations in the magnitude of E_0_, in the amplitude of interannual variability, and in long-term trends. The absolute E_0_ magnitudes are different between GFDL-ESM2M and CanESM2, with values for CanESM2 substantially much greater, in particular when estimated using the Penman-Monteith and Hargreaves-Samani formulations. The warm and dry bias in CanESM2 is expected to cause this difference. Despite these differences between the two GCMs, we find that the Penman-Monteith-based E_0_ estimation is both higher in magnitude of mean seasonal totals as well as the amplitude of interannual variability relative to the other two formulations. Priestley-Taylor exhibits the lowest interannual variability, similar to results found for coefficient of variation in Fig 3. Because both Priestley-Taylor and Hargreaves-Samani estimations yield overall lower magnitudes for climatological mean and amplitude of interannual variability in E_0_, they have a large influence on the trends in percent of historical mean and standardized anomaly (Fig 5, bottom two rows), particularly the latter. For example, in the plots for GFDL-ESM2M (left column), Priestley-Taylor exhibits a comparably low E_0_ climatology (similar to Hargreaves-Samani) and the lowest interannual variability, yielding the largest 21^st^ century trends in E_0_ in both percent of historical mean and standardized anomalies.

Our analysis of E_0_ responses across the three formulations shows that Penman-Monteith produces a more spatially heterogeneous response in E_0_ as well as a more enhanced realization of the amplitude of interannual variability. We believe that both these features are a result of a more adequate treatment of wind-driven turbulence and relative humidity in Penman-Monteith, and therefore this formulation is particularly critical for regions, such as the southwestern US and the Great Plains, that are sensitive to these drivers. In the next section, we focus on just the Penman-Monteith formulation and examine how the E_0_ responses are further sensitive to the choices of input variables representing both the aerodynamic and radiative terms.

### Penman-Monteith E_0_ responses based on the choice of input parameters

**Fig 6:**
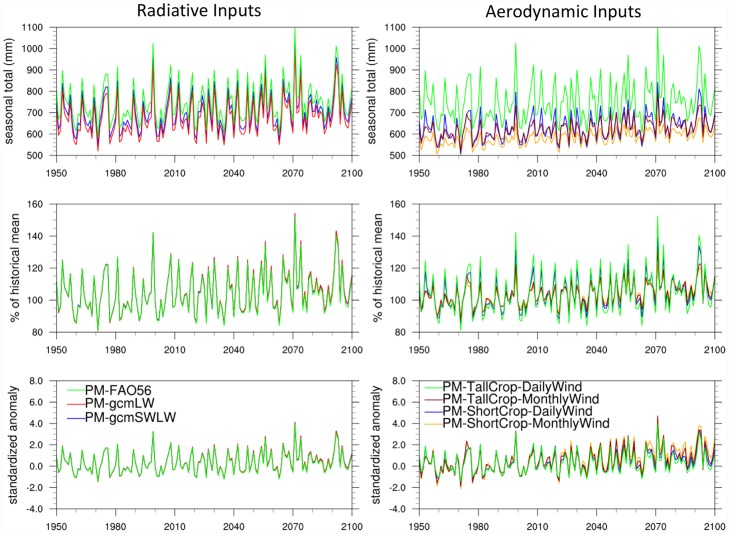
Same as Fig 5 but using only the Penman-Monteith formulation and variations made to (a) radiation inputs (left column): Strict FAO56 with fixed albedo (0.23) and parameterized longwave radiation (green), fixed albedo and GCM-modeled longwave radiation (red), and GCM-modeled net shortwave and longwave radiation (blue); and (b) to crop selection and/or daily versus monthly timescales for wind speed (right column): Tall reference crop and daily wind (green), tall reference crop and monthly wind (brown), short reference crop and daily wind (blue), short reference crop and monthly wind (yellow). Analysis based on GFDL-ESM2M only.

We first examine the sensitivity of projected E_0_ trends to different choices of radiative inputs into the Penman-Monteith formulation. In Fig 6 (left column), we compare the following three cases for this analysis, using data from the GFDL-ESM2M model only: (a) fixed surface albedo (0.23) and parameterized longwave radiation balance based on FAO56, (b) fixed surface albedo (0.23) based on FAO56 but GCM-modeled longwave radiation balance, and (c) GCM-modeled net shortwave and longwave radiation balances. For the Northern Great Plains, the use of parameterized versus model’s longwave creates the largest difference for absolute values of E_0_. The choice of fixed versus modeled variable albedo has very little effect on the trends. Overall, the radiative input choices have an extremely limited effect on differentiating the E_0_ timeseries in percent of mean and standardized E_0_ anomalies during the 21^st^ century.

Next, we examined the sensitivity of projected E_0_ responses to input of wind speed and crop height, which appear in the aerodynamic term. Fig 6 (right column) shows MJJAS E_0_ trends for the Northern Great Plains for differences in the aerodynamic term due to (i) modeling tall versus short reference crops, and (ii) using daily wind speeds versus monthly wind speeds averaged from daily values. In general, the results show that (a) tall crops estimate E_0_ roughly 20% higher than short crops, and (b) the use of daily wind speed also yields higher E_0_ than for monthly wind speed, although the magnitude of that difference depends on the crop type (surface roughness). Furthermore, interannual variability is higher for daily wind speed and tall crop type. These factors also affect 21^st^ century trends in percent of historical mean and standardized anomalies, however differences due to the choice of input parameters are much smaller relative to the differences found between the three different E_0_ formulations in the previous section (see Fig 5). Although in Fig 6 we only show the sensitivity of Penman-Monteith formulation to choices of radiative and advective terms based on GFDL-ESM2M, we find similar results with CanESM2.

### Sensitivity of 21^st^ century projections of drought indices to E_0_ formulation and GCM selection

One approach to explore changing drought risk under climate change is to evaluate future projections of currently used drought indices. Here, we examine 21^st^ century projections for the Northern Great Plains domain in two drought indices: the Evaporative Demand Drought Index (EDDI; [36,66]), and the Standardized Precipitation Evapotranspiration Index (SPEI; [65]), as a function of E_0_ formulation and climate model selection. As mentioned in the Methods section, the SPEI index calculated and plotted here uses the same standardization method as used in calculating EDDI in order to better compare the EDDI and SPEI projections. However, for EDDI, positive values denote drier conditions, whereas for SPEI, negative values denote drier conditions. We compare the projections of these indices between GFDL-ESM2M and CanESM2 which have different biases and climate sensitivities. GFDL-ESM2M has a cold/wet bias for the Northern Great Plains, where CanESM2 has a hot/dry bias in the historical period. Furthermore, CanESM2 has higher global climate sensitivity to CO_2_ increase than GFDL-ESM2M. Given these differences, we would expect CanESM2 to yield EDDI and SPEI indices that project a more drought-prone future for the Northern Great Plains, relative to GFDL-ESM2M.

**Fig 7:**
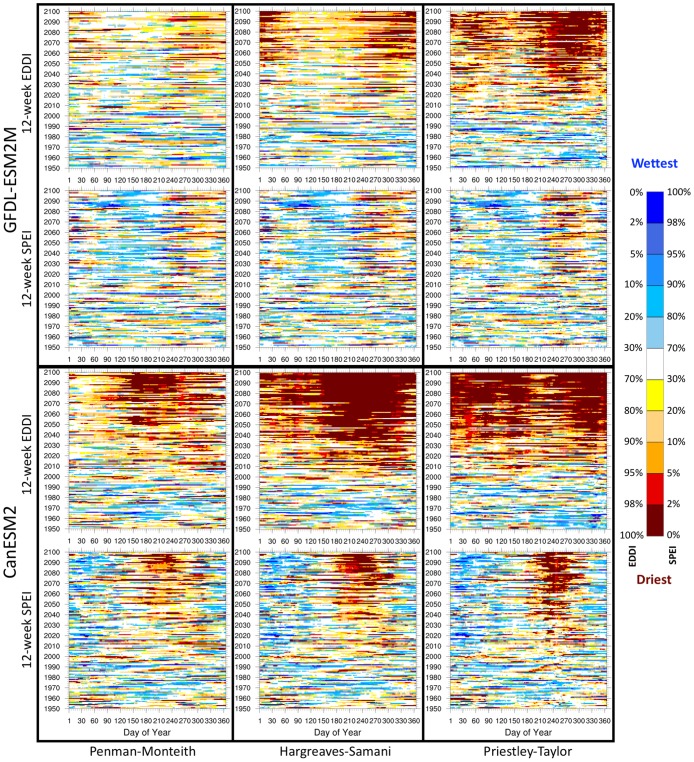
21^st^ century trends in EDDI and SPEI. Comparison of 12-week EDDI and SPEI computed with the three E_0_ formulations for each day between 1950–2100 for GFDL-ESM2M and CanESM2 for the Northern Great Plains region. Daily EDDI or SPEI values are binned into specific percentile categories (spanning between driest and wettest categories) relative to the historical (1976–2005) distribution.

Fig 7 shows projections of 12-week EDDI and SPEI between 1950–2100 for the two GCMs. Each day’s (Day of Year, *x* axis within each plot) projected EDDI or SPEI value is binned into a specific percentile category (spanning between driest and wettest categories) relative to the historical (1976–2005) distribution. For GFDL-ESM2M, the primary difference is found in EDDI projections across the three E_0_ formulations. Priestley-Taylor shows the strongest tendency for dry conditions throughout the 21^st^ century. Hargreaves-Samani shows a similar overall pattern to Priestley-Taylor, although the magnitude of the drying response is comparatively lower. Penman-Monteith shows the smallest trend for increases in the 21^st^ century drought risk, but still shows a positive drying trend in the late summer and fall period. On the other hand, the SPEI plots look very similar across the three E_0_ formulations, and closer to the EDDI plot based on Penman-Monteith. For CanESM2, EDDI projections also differ much more across the three formulations relative to the SPEI plots. But for both indices, Hargreaves-Samani and Priestley-Taylor show a more pronounced drying tendency relative to Penman-Monteith.

Both GCMs show an increased tendency for droughts (as defined during the historical period) during the 21^st^ century, although there are large differences based on the choice of E_0_ formulation and GCM. The drought tendency is much more pronounced for EDDI than SPEI across all formulations and models, however the difference between EDDI and SPEI plots is smallest for Penman-Monteith. Overall, CanESM2 projects a more drought-prone future compared to GFDL-ESM2M. A seasonal cycle is also apparent with increased cold-season drought projected in GFDL-ESM2M and increased warm-season drought in CanESM2. We expect that the difference between the EDDI and SPEI responses are primarily because, at any given time, EDDI is only sensitive to the evaporative demand (E_0_) while SPEI is responding to the difference of moisture supply and evaporative demand (P—E_0_). Therefore, trends and model bias in precipitation will affect the SPEI responses. An increasing trend in precipitation, which is found for both models, can lessen or counteract a trend toward increasing dryness in SPEI. A positive trend in precipitation can also dampen the increase in E_0_ through the complementary relationship [89,90] by increasing available moisture, thereby impacting EDDI. Another reason the future SPEI trends are weaker than the EDDI trends has to do with the fact that precipitation is much more variable than E_0_. In other words, P-E_0_ has a much wider distribution in the historic period than E_0_ itself. Therefore, when standardized relative to the historical distribution, future E_0_ trends will appear more augmented than future P-E_0_ trends, especially if future P trends are relatively small.

GCM precipitation bias during the historical period can impact these drought indices in two ways. First, a cold and wet bias as well as lower climate sensitivity for temperature in GFDL-ESM2M strengthens P and attenuates E_0_, while the opposite is true for CanESM2, which has a hot and dry bias. Second, a hot and dry bias will make EDDI more sensitive to future climate warming as it increases the aridity of the model’s historical climate. An arid climate is more prone to conditions where the surface evapotranspiration cannot meet E_0_ leading to more enhanced E_0_ (and therefore, EDDI) because of soil moisture-temperature feedbacks that amplify surface temperature and water vapor deficit (i.e., the basis of the complementary relationship [90]). A more detailed investigation of the relationship between GCM bias and drought projections is a topic of future research, as regional temperature and precipitation biases inherent in GCMs can strongly influence the trends in the 21^st^ century projections of these drought indices. Appropriate bias-corrected GCM datasets could help in reducing the uncertainty in the projection of regional drought risks.

## Conclusions

In this study we evaluated how different methodological and dataset choices in the estimation of evaporative demand yield different assessments of temporal and spatial variability in E_0_ and trends in 21^st^ century drought risk. First, we evaluated these differential responses across three of the more widely used formulations for E_0_ estimations: Penman-Monteith, Hargreaves-Samani, and Priestley-Taylor. Our analysis shows that Penman-Monteith provides a more physically robust treatment of E_0_ across the CONUS. This formulation gives a more spatially heterogeneous realization of E_0_ because it is more sensitive to the variations in turbulent fluxes (driven by wind speed and surface roughness) and relative humidity. Penman-Monteith also facilitates a more physically robust depiction of the amplitude of interannual variability relative to the other two methods, whose interannual variabilities are much lower, particularly Priestley-Taylor. We also find regional patterns to this enhanced interannual variability in Penman-Monteith, such as in the Great Plains and southwestern US, which is corroborated by pan evaporation observations. This feature is not reproduced by Hargreaves-Samani and Priestley-Taylor. Previous studies have also shown a greater importance of drivers like wind speed and relative humidity in these regions in driving interannual variability in E_0_ [5,72,88]. Therefore, the application of Penman-Monteith for E_0_ estimation would especially be critical for regions where the influence of these drivers is strong.

We also find that Penman-Monteith is more appropriate for 21^st^ century E_0_ projections and for drought indices that use E_0_, e.g., EDDI and SPEI. The 21^st^ century E_0_ trends generated in our analysis, particularly trends in percent of historical mean and standardized anomalies, are found to be sensitive to the long-term mean value and the amplitude of interannual variability; i.e. if the magnitude of E_0_ and its interannual variability are relatively low for a particular E_0_ formulation, then the normalized or standardized 21^st^ century trend based on that formulation is more amplified relative to other formulations. We find this to be the case with the Hargreaves-Samani and Priestley-Taylor formulations, where the future standardized E_0_ trends are much larger than those from Penman-Monteith.

In examining the Penman-Monteith-driven E_0_ response under various input variable choices related to radiation, surface roughness, and wind speed, we found differences in E_0_ trends. However, these differences are much smaller than the differences in E_0_ trends from the three E_0_ formulations. When making a choice for crop type, which determines surface roughness, we find that parameterizing for tall crop leads to higher (by about 20%) long-term mean E_0_ and interannual variability, which ultimately impacts the projected standardized anomalies. We find that the use of the wind speed term averaged across different temporal scales (daily versus monthly) has a similar effect on both the estimation of long-term mean E_0_ value and interannual variability, i.e., monthly timescales lead to lower mean E_0_ and interannual variability than daily timescales, but the magnitude of that effect varies with crop type. We also found that different choices in estimating the radiation term have a very limited influence on E_0_ trends into the 21^st^ century.

**Fig 8:**
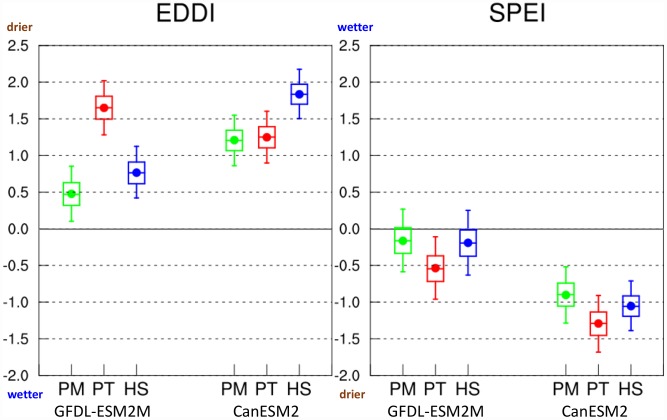
Uncertainties in changing drought risk by 2050 based on E_0_ formulation and GCM selection. Comparison of changes in 12-week EDDI and SPEI values for August 31 by 2050 (relative to the 1976–2005 mean) between the two GCMs based on the different E_0_ formulations considered in this study. Filled circles show the mean change, box plots show 25th, 50th and 75th percentiles, and whiskers show 5th and 95th percentiles. Confidence intervals shown here about the mean projected change are estimated based on Monte Carlo resampling. Positive changes in EDDI and negative changes in SPEI signify increases in drought intensity.

The choice of E_0_ formulation and specific GCM selection also have a large influence on the projected changes in standardized drought indices as shown by Fig 8, which shows projected changes in summertime (12-week, August 31) EDDI and SPEI by 2050 for the different E_0_ formulations and GCMs evaluated in this study. In the absence of an adequate number of GCM ensemble member runs available, we performed a Monte Carlo resampling (with replacement) of the data to provide uncertainty ranges for these estimates (5th to 95th percentiles). Positive changes in EDDI and negative changes in SPEI imply increases in drought intensity. Depending on the E_0_ formulation and GCM selection, we see a large range in future changes in these indices, yet all results point to increases in drought conditions by 2050, except for the lower 25 percentile cases in GFDL-ESM2M for SPEI based on Penman-Monteith and Hargreaves-Samani. Overall, the use of Penman-Monteith formulation leads to more moderate increases in future drought risk while, for the most part, Priestley-Taylor causes the largest increases in drought risk, and Hargreaves-Samani falls between the two. As expected, the use of data from CanESM2, which has higher climate sensitivity than GFDL-ESM2M as well as a hot and dry bias, leads to a greater drought risk than GFDL-ESM2M.

We have demonstrated here that methodological and dataset choices are crucial to the assessment of 21^st^ century drought risk under climate change. Based on our findings, we would recommend a revaluation of results from previous studies that are based on projected trends in the standardized drought parameters. Furthermore, biases in GCMs affect the quantification of projected changes in evaporative demand and the drought indices based on it, introducing additional uncertainty. Use of physically consistent bias corrected and downscaled GCMs output at daily timescales will help to avoid the problem of GCM bias when exploring future drought risks across a suite of GCMs. Future studies examining trends in 21^st^ century drought risk should also attempt to incorporate the effects of increased water-use efficiency of plants at elevated CO_2_ levels on E_0_ in order to not overestimate the drying risk. Overall, an appropriate selection of methodology and dataset for E_0_ estimation based on these findings will facilitate a better assessment of future drought risk.

## Supporting information

S1 Table Stations from the NOAA cooperative observer network with pan evaporation data used to extend the dataset originally compiled by Hobbins (2004). (DOCX)
