# Supplementary material for: Drought risk assessment under climate change is sensitive to methodological choices for the estimation of evaporative demand
Source: PLoS One. 2017 Mar 16;12(3):e0174045. doi: 10.1371/journal.pone.0174045 (PMC5354442; doi:10.1371/journal.pone.0174045)
Supplement: S1 Table — (DOCX) [file pone.0174045.s001.docx]

S1 Table. Stations from the NOAA Cooperative Observer Network with pan evaporation data used to extend the dataset originally compiled by Hobbins (2004).

| **COOPID** | **NAME** | **STATE** |
| --- | --- | --- |
| 105275 | LIFTON PUMPING STN | ID |
| 106152 | MOSCOW U OF I | ID |
| 204502 | LAKE CITY EXP FARM | MI |
| 218692 | WASECA S RSCH & OU | MN |
| 241470 | CANYON FERRY DAM | MT |
| 243110 | FT ASSINIBOINE | MT |
| 244345 | HUNTLEY EXP STN | MT |
| 255590 | MITCHELL 5E | NE |
| 325479 | MANDAN EXP STN | ND |
| 329430 | WILLISTON EXP FARM | ND |
| 351902 | COTTAGE GROVE DAM | OR |
| 355142 | MADRAS 2 N | OR |
| 359316 | WICKIUP DAM | OR |
| 391972 | COTTONWOOD 2 E | SD |
| 425194 | LOGAN 5 SW EXP FAR | UT |
| 452542 | ELTOPIA 8 WSW | WA |
| 454679 | LIND 3 NE | WA |
| 459200 | WHITMAN MISSION | WA |
| 484411 | HEART MTN | WY |
| 487105 | PATHFINDER DAM | WY |
| 488160 | SHERIDAN FLD STN | WY |

Hobbins MT. 2004. Regional evapotranspiration and pan evaporation: complementary interactions and long-term trends across the conterminous United States [dissertation]. Fort Collins (CO): Colorado State University.
